# Mesenchymal stromal cells from *JAK2* ^*V617F*^ myeloproliferative neoplasms support healthy and malignant hematopoiesis in a humanized scaffold model in vivo

**DOI:** 10.1002/hem3.70185

**Published:** 2025-08-22

**Authors:** Alessandra Ferrelli, Syed Mian, Marion Piganeau, Hector Huerga Encabo, Despoina Papazoglou, Giuseppe D'Agostino, Fatihah Mohamad Nor, Manuel Garcia‐Albornoz, Steven Ngo, Linda Ariza‐McNaughton, Erika Morsia, Matteo Giovanni Della Porta, Claire Harrison, Shahram Kordasti, Dominique Bonnet

**Affiliations:** ^1^ Haematopoietic Stem Cell Laboratory The Francis Crick Institute London UK; ^2^ School of Cancer and Pharmaceutical Sciences King's College London London UK; ^3^ Plasticell Limited Stevenage Bioscience Catalyst Stevenage UK; ^4^ Department of Clinical and Molecular Sciences Università Politecnica della Marche Ancona Italy; ^5^ Department of Biomedical Sciences Humanitas University Milan Italy; ^6^ IRCCS Humanitas Research Hospital Milan Italy; ^7^ Haematology Department Guy's Hospital London UK

## Abstract

Myeloproliferative Neoplasms (MPN) are malignancies of hematopoietic stem and progenitor cells (HSPCs) that lead to the overproduction of mature blood cells. These disorders include Essential Thrombocythemia (ET), Polycythemia Vera (PV), and Primary Myelofibrosis (PMF), primarily driven by somatic mutations such as *JAK2* ^*V617F*^. Research indicates that mesenchymal stromal cells (MSCs) support fibrosis in PMF, though their role in ET and PV remains less clear. Furthermore, in vivo studies of ET/PV HSPCs remain a challenge due to low engraftment levels in xenograft models. We employed a 3D scaffold model to create an MPN humanized xenograft mouse model, enabling in vivo functional studies of primary MPN progenitor cells and the supportive role of human MSCs. Using this model, we first demonstrated robust hematopoietic support of healthy (HD) HSPCs by PV and ET MSCs. We then investigated the role of MSCs in sustaining *JAK2* ^*V617F*^ mutant cells by using a CRISPR‐Cas9 editing model, along with primary PV and ET HSPCs. Our results showed consistent engraftment of CRISPR‐edited *JAK2* ^*V617F*^ mutant HSPCs and PV and ET patient‐derived HSPCs in scaffolds seeded with HD, PV, and ET stroma, providing the first in vivo evidence that PV and ET MSCs can sustain both healthy and MPN‐associated hematopoiesis. Furthermore, HD MSCs were also capable of sustaining PV and ET HSPCs in vivo. Overall, we present the first humanized MPN xenograft model that offers valuable insights into how human BM MSCs interact with *JAK2* ^*V617F*^ mutant clones.

## Graphical abstract

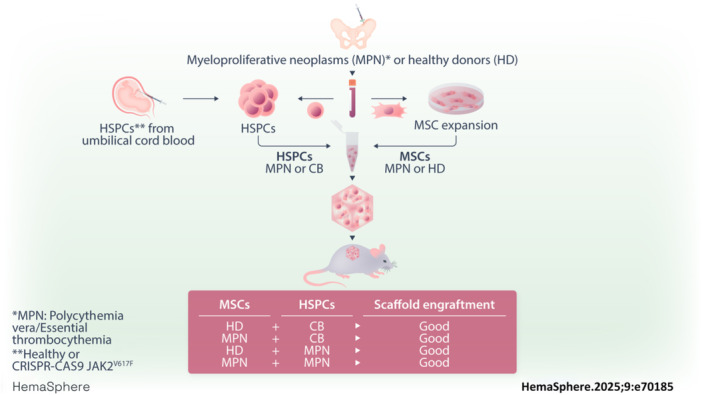

## INTRODUCTION

Myeloproliferative neoplasms (MPN) are hematopoietic stem and progenitor cells (HSPCs) malignancies, characterized by the excessive production of mature blood cells, including red blood cells (RBCs) and platelets. These neoplasms include Essential Thrombocythemia (ET), Polycythemia Vera (PV), and Primary Myelofibrosis (PMF).^1^ ET is marked by an overproduction of megakaryocytes, PV by an abundance of erythrocytes, and PMF by the development of bone marrow (BM) fibrosis as well as extramedullary hematopoiesis.^1^ The pathogenesis of MPN is primarily driven by three major mutations, with the *JAK2* ^*V617F*^ mutation being the most prevalent.^2^ It has been suggested that the hematopoietic stem cell (HSC) BM microenvironment plays a crucial role in regulating both normal and stress‐induced hematopoiesis.^3^, ^4^ Increasing evidence suggests that in myeloid malignancies, the BM microenvironment supports the survival and proliferation of mutant clones.^5^, ^6^

Previous research, particularly on myelofibrosis (MF), has demonstrated that mesenchymal stromal cells (MSCs) undergo significant phenotypic changes, contributing to fibrosis, inflammation, and disease progression.^7^, ^8^, ^9^ MSCs derived from MF patients are reprogrammed at both transcriptomic and functional levels.^10^ However, little is known about MSCs from PV and ET patients. These disorders exhibit distinct phenotypes, clinical symptoms, and progression patterns compared to MF, and the hematopoietic‐supporting role of MSCs in PV and ET remains unknown.

The study of MPN pathogenesis has often relied on murine models, such as retroviral transduction mouse models^11^, ^12^ and genetically engineered systems.^13^, ^14^ Xenograft models have been used primarily in MF due to the poor engraftment levels of PV and ET HSPCs, which require large numbers of cells, adding practical challenges to experimental studies.^15^, ^16^, ^17^, ^18^ Moreover, these models fall short in replicating the complexity of the human BM microenvironment, particularly due to the absence of human stroma, emphasising the need for humanized in vivo models of MPN.

A more detailed exploration of MSCs from PV and ET patients could provide crucial insights into the intricate cellular interactions within their specialized microenvironments. This deeper understanding may reveal previously unknown mechanisms driving disease initiation and progression, as well as therapeutic resistance, offering new opportunities for targeted interventions.

## METHODS

### Umbilical cord blood (UCB) cells

UCB samples were obtained from Royal London Hospital (London, UK) after normal full‐term births. Informed and written consent was obtained prior to the collection, in accordance with procedure approved by the East London and Research Ethics Committee.

Mononuclear cells (MNCs) were isolated by density centrifugation using Ficoll‐Paque (GE 67 Healthcare). CD34^+^ HSPCs were positively selected with EasySep Human CD34 Positive Selection Kit (Cat 18056; Stem Cell Technologies) according to the manufacturer's instructions.

### CRISPR‐Cas9 *JAK2* ^*V617F*^ editing

For genome editing of primary human HSPCs we follow our previous protocol.^19^ Isolated CB CD34^+^ HSPCs were kept in culture in Stem span SFEM II (Cat 09605; Stem Cell Technologies) supplemented with rhSCF, rhFLT3‐L, and rhTPO at 100 ng/mL (Cat 300‐07, Cat 300‐19, Cat 300‐18, respectively; Peprotech). After ~36 h, cells were collected and CRISPR editing performed using the NEON Transfection system (Cat MPK1025; Thermofisher). Small guide RNA and donor DNA template are listed in Supporting Information S1: Table 5. After another 48 h, cells were counted and used for either in vitro culture or pre‐seeded in gelfoam sponges prior to subcutaneous implantation in immunodeficient mice. CRISPR efficiency was also analyzed by next generation sequencing at Day 0.

### In vitro co‐culture of CB CD34^+^ HSPCs with HD and MPN MSCs

HD, PV or ET MSCs were seeded at a density of 5 × 10^5^ cells/well in a 12‐well plate and cultured overnight. CRISPR‐edited CD34^+^ HSPCs (1–2 × 10^5^) were added the day after in Myelocult H5100 (Cat 05150; Stem Cell Technologies) containing a cocktail of cytokines (3GT = 20 ng/mL G‐CSF, 20 ng/mL IL‐3, 20 ng/mL TPO, Peprotech, Cat 300‐23, Cat 200‐03, Cat 300‐18, respectively). After 1 week and 2 weeks, hCD45^+^ cells were isolated performing CD45 selection kit (EasySep Human CD45 kit II, Cat 18259; Stem Cell Technologies), following manufacturer's instructions. As inflammation challenge, 1 µg/ml of LPS derived from Escherichia coli strain 0111: B4 (L4391, Sigma) and 1 ng/ml of IL‐1β (200‐01B) were used.

### Targeted DNA mutation sequencing

Targeted mutational analysis was performed on CRISPR‐edited cells and xenografted cells. Primers used to assess *JAK2* ^*V617F*^ mutation burden are listed in Supporting Information S1: Table 5. MiSeq targeted sequencing was performed and FASTQ files were then analyzed using IGV_2.8.9 software (Human HG19 as genome reference). The point mutation percentage of the target sequence was manually annotated.

### Patient samples

**Figure 1:**
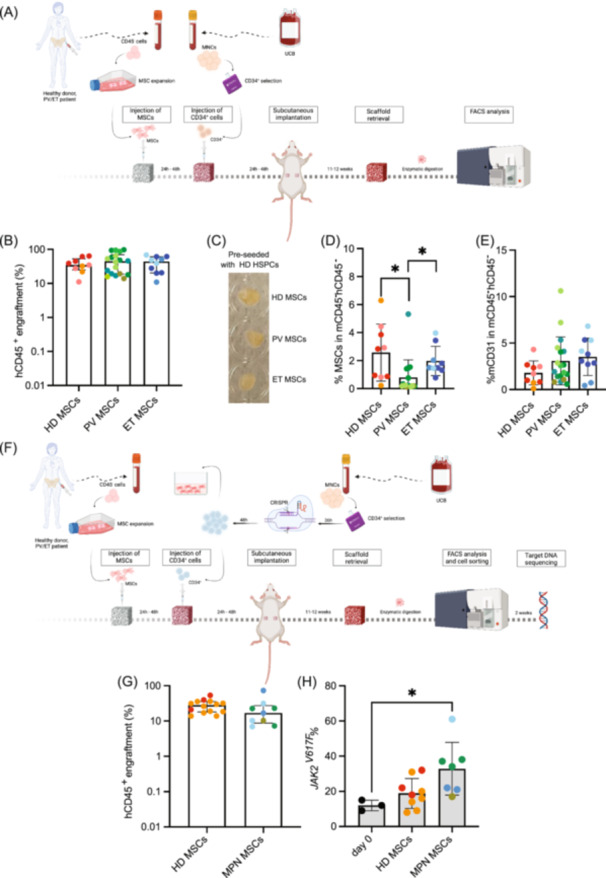
**PV and ET BM‐patient‐derived MSCs can support healthy and CRISPR‐edited cord blood CD34** ^**+**^ **HSPCs**. **(A)** Schematic representation of the protocol used to generate humanized scaffolds with HD, PV, or ET BM patient‐derived MSCs and HD CD34^+^ HSPCs. Created with Biorender. **(B)** hCD45^+^ engraftment in scaffolds pre‐seeded with either HD, PV, or ET MSCs and healthy hCD34^+^ HSPCs after 11–12 weeks of scaffold implantation in NSG‐SGM3 mice. Data representative of three independent experiments, each performed with hCD34^+^ HSPCs from different human umbilical cord blood donors. Same donor/patient MSCs are color coded. Each dot represents one scaffold. Error bars: median ± interquartile range. Ordinary one‐way ANOVA test used for significance (nonsignificant). **(C)** Photo of representative scaffolds pre‐seeded with HD, PV, or ET MSCs and HD CD34^+^ HSPCs and retrieved at 12 weeks from in vivo implantation. **(D)** Percentage of MSCs (mCD45^−^hCD45^−^ hCD73^+^hCD90^+^) in the mCD45^–^hCD45^–^ population at the end of the scaffold experiment (scaffolds from Figure 1B). Kruskal–Wallis test was performed to assess statistical significance. **(E)** Percentage of murine endothelial cells (mCD45^–^hCD45^–^mCD31^+^) in the mCD45^–^hCD45^–^ population at the end of the scaffold experiment (scaffolds from Figure 1B). Kruskal–Wallis test was performed to assess statistical significance (nonsignificant). **(F)** Schematic representation of the protocol used to generate CRISPR‐edited CD34^+^ HSPCs presenting *JAK2* ^*V617F*^ mutation. These cells were then used to be pre‐seeded in humanized scaffolds with HD, PV, or ET BM patient‐derived MSCs or in ex vivo co‐cultures with HD, PV, or ET BM patient‐derived MSCs as stromal layer. Created with Biorender. **(G)** hCD45^+^ engraftment in scaffolds pre‐seeded with either HD or MPN MSCs and CRISPR‐edited hCD34^+^ HSPCs after 11–12 weeks of scaffold implantation in NSG‐SGM3 mice. Each dot represents one scaffold. Error bars: median ± interquartile range. Same donor MSCs are color coded (red shades are used for HD‐MSCs, blue shades are used for ET MSCs, while green shades are used for PV MSCs). **(H)** Comparison of the VAF between the *JAK2* ^*V617F*^ mutation burden at Day 0 in CRISPR‐edited CD34^+^ HSPCs (beginning of the in vivo experiment) and the *JAK2* ^*V617F*^ mutation burden at end of the in vivo experiment in hCD45^+^ cells in HD MSC pre‐seeded or MPN MSC pre‐seeded scaffolds. Error bars: mean ± SD. Same donor MSCs are color coded (red shades are used for HD‐MSCs, blue shades are used for ET MSCs, while green shades are used for PV MSCs). Kruskal–Wallis test used for significance (*p = 0.0332).

**Figure 2:**
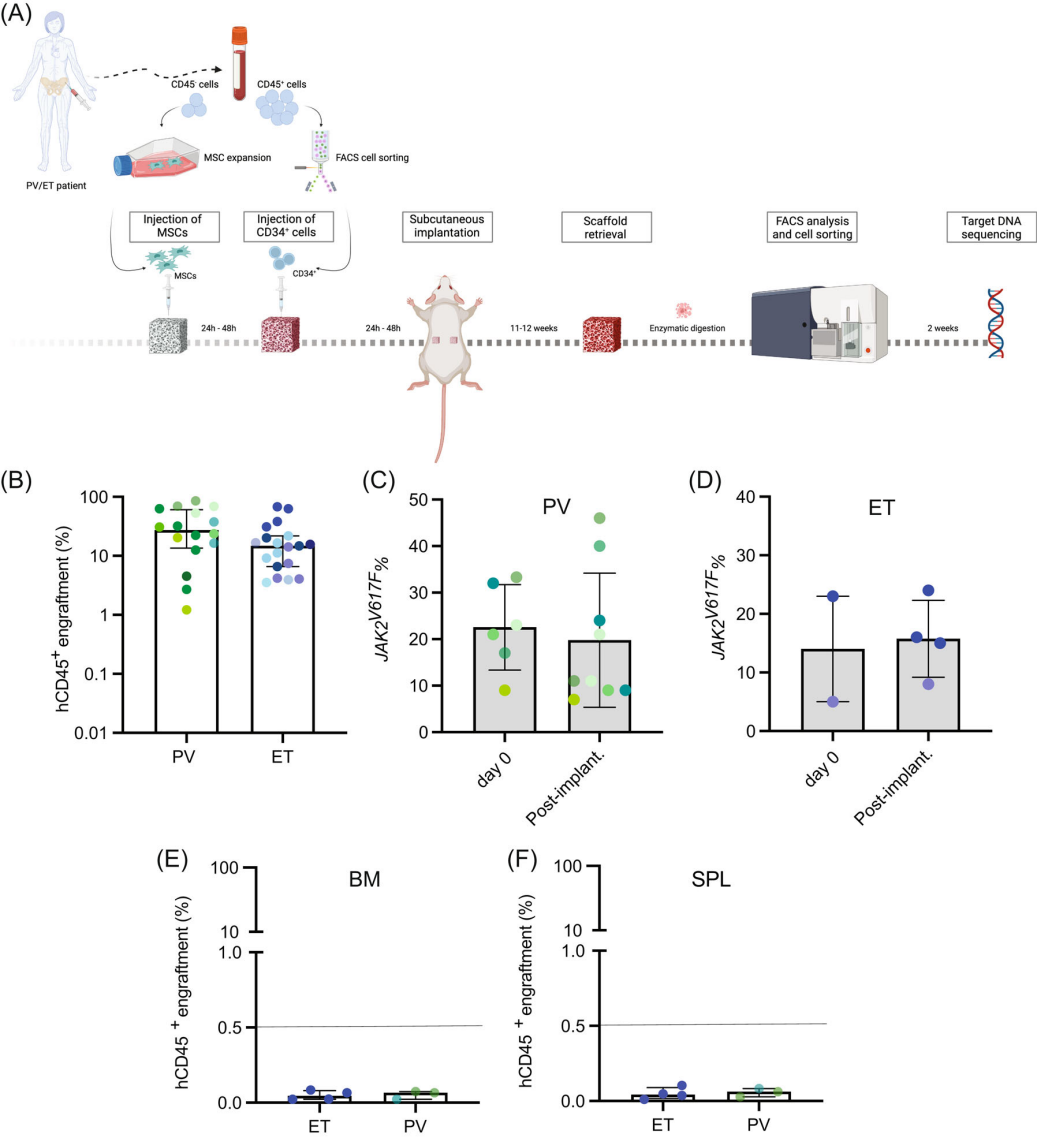
**ET and PV BM patient‐derived CD34** ^**+**^ **HSPCs engraft in the 3D scaffold humanized model in vivo**. **(A)** Schematic representation of the protocol used to generate humanized scaffolds with ET or PV BM patient‐derived MSCs and ET and PV BM patient‐derived CD34^+^ HSPCs, respectively. Created with Biorender. **(B)** hCD45^+^ engraftment in scaffolds pre‐seeded with either PV or ET MSCs and PV or ET hCD34^+^ HSPCs after 11–12 weeks of scaffold implantation in NSG‐SGM3 mice. For PV scaffolds, 14 patient samples were used: 5 patient samples were used singularly, while the rest was used by pooling CD34^+^ HSPCs of different patient samples (3 pools generated). PV MSCs were used either as single patient MSCs or as a pool of 3 patient MSCs. For ET scaffolds, 18 patient samples were used: 4 patient samples were used singularly, while the rest was used by pooling CD34^+^ cells of different patient samples (4 pools generated). Each dot represents one scaffold. Each pool of patient CD34^+^ HSPCs is color coded. Error bars: median ± interquartile range. Mann–Whitney test was performed for statistical significance (non‐significant). **(C)** Comparison of the VAF between the *JAK2* ^*V617F*^ mutation burden at Day 0 in CD34^+^ cells (beginning of the in vivo experiment) and the *JAK2* ^*V617F*^ mutation burden at 12 weeks post‐scaffold implantation in vivo in hCD45^+^ cells in PV scaffolds. Each pool of patient CD34^+^ HSPCs are color coded. Error bars: mean ± SD. Mann–Whitney test was performed to assess statistical significance (nonsignificant). **(D)** Comparison of the VAF between the *JAK2* ^*V617F*^ mutation burden at Day 0 in CD34^+^ HSPCs (beginning of the in vivo experiment) and the *JAK2* ^*V617F*^ mutation burden at 12 weeks post‐scaffold implantation in vivo in hCD45^+^ cells in ET scaffolds. Each pool of patient CD34^+^ HSPCs are color coded. Error bars: mean ± SD. Paired *t* test was performed to assess statistical significance (non‐significant). **(E)** hCD45^+^ engraftment in the bone marrow (BM) of NSG‐SGM3 mice implanted with PV and ET scaffolds. Each dot represents one mouse. Each pool of patient CD34^+^ HSPCs is color coded. *N* = 6. Error bars: median ± interquartile range. **(F)** hCD45^+^ engraftment in the spleen (SPL) of NSG‐SGM3 mice implanted with PV and ET scaffolds. Each dot represents one mouse. Each pool of patient CD34^+^ HSPCs is color coded. *N* = 6. Error bars: median ± interquartile range.

**Figure 3:**
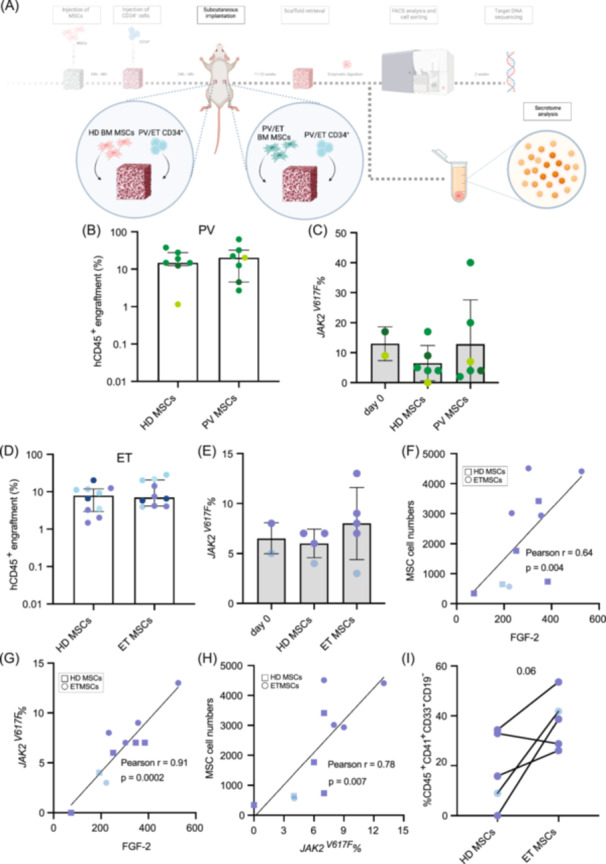
**PV and ET CD34** ^**+**^ **HSPCs can be sustained by both HD and PV or ET MSCs, respectively in a humanized in vivo model. (A)** Schematic representation of the experimental design used to generate humanized scaffolds with HD, PV, or ET BM patient‐derived MSCs and PV or ET BM patient‐derived CD34^+^ HSPCs, respectively. Created with Biorender. **(B)** hCD45^+^ engraftment in scaffolds pre‐seeded with either HD or PV MSCs and PV hCD34^+^ HSPCs after 11–12 weeks of scaffold implantation in NSG‐SGM3 mice. 17 patient samples were used: 3 patient samples were used singularly, while 14 samples were used by pooling CD34^+^ HSPCs (3 pools generated). Each dot represents one scaffold. Each pool of patient CD34^+^ HSPCs are color coded. Error bars: median ± interquartile range. Wilcoxon test was performed to test for statistical significance (nonsignificant). **(C)** Comparison of the VAF between the *JAK2* ^*V617F*^ mutation burden at Day 0 in CD34^+^ HSPCs (beginning of the in vivo experiment) and the *JAK2* ^*V617F*^ mutation burden at 12 weeks post‐scaffold implantation in vivo in hCD45^+^ cells in PV scaffolds pre‐seeded with either HD or PV MSCs. Each pool of patient CD34^+^ HSPCs are color coded. Error bars: mean ± SD. **(D)** hCD45^+^ engraftment in scaffolds pre‐seeded with either HD or ET MSCs and ET hCD34^+^ HSPCs after 11–12 weeks of scaffold implantation in NSG‐SGM3 mice. Three pools of patient sample derived hCD34^+^ HSPCs were used. Each dot represents one scaffold. Error bars: median ± interquartile range. Each pool of patient CD34^+^ HSPCs is color coded. Wilcoxon test was performed to test for statistical significance (nonsignificant). **(E)** Comparison of the VAF between the *JAK2* ^*V617F*^ mutation burden at Day 0 in CD34^+^ HSPCs (beginning of the in vivo experiment) and the *JAK2* ^*V617F*^ mutation burden at 12 weeks post‐scaffold implantation in vivo in hCD45^+^ cells in ET scaffolds pre‐seeded with either HD or ET MSCs. Each pool of patient CD34^+^ HSPCs is color coded. Error bars: mean ± SD. **(F)** Correlation between MSC cell numbers retrieved from ET scaffolds and FGF‐2 release in the scaffolds. Each pool of patient CD34^+^ HSPCs is color coded. Significant correlation was measured by linear regression analysis. **(G)** Correlation between *JAK2* ^*V617F*^ mutation burden in ET scaffolds and FGF‐2 release in the scaffolds. Each pool of patient CD34^+^ HSPCs is color coded. Significant correlation was measured by linear regression analysis. **(H)** Correlation between *JAK2* ^*V617F*^ mutation burden and MSC cell numbers retrieved in ET CD34^+^ HSPCs pre‐seeded scaffolds. Each pool of patient CD34^+^ HSPCs are color coded. Significant correlation was measured by linear regression analysis. **(I)** Percentage of hCD45^+^CD41^+^CD33^−^CD19^−^ cells in scaffolds implanted with ET CD34^+^ HSPCs pre‐seeded with either HD or ET MSCs. Each line of connected dots represents one humanized mouse. Each pool of patient CD34^+^ HSPCs is color coded. Paired *t* test was performed to test significance.

MPN BM samples were collected at diagnosis from Guy's Hospital under the research ethics approval 12‐EE‐0493. Frozen MPN BM samples were also received by Humanitas Research Hospital, Milan, Italy, and Università Politecnica delle Marche, AOU delle Marche, Ancona, Italy, covered under material transfer agreements. Overall list of patient samples used can be found in Supporting Information S1: Table 1. Samples used in each specific figure can be found in Supporting Information S1: Table 2 (for samples used in Figure 1), Supporting Information S1: Table 3 (for samples used in Figure 2) and Supporting Information S1: Table 4 (for samples used in Figure 3).

### MSC expansion

MNCs were isolated by density centrifugation using Ficoll‐Paque (GE 67 Healthcare). To separate CD45^+^ and CD45^−^ cells, CD45 selection kit (EasySep Human CD45 kit II, Cat 18259; Stem Cell Technologies) was used by following manufacturer's instructions. CD45^+^ cells were then frozen for future experiments, while CD45^−^ cells were seeded at a density of 1 × 10^6^/cm^2^/0.2 to 0.3 mL of MSC culture media. MSCs were expanded and cultured in MEM Alpha Medium (1X) + GlutaMAX‐1 (Cat 32571‐029; Gibco‐Life Technologies) supplemented with 10% hMSC‐specific fetal bovine serum (FBS) (Cat 12662‐029; Gibco‐Life Technologies) and 1% penicillin/streptomycin (Cat P4333; Sigma‐Aldrich). MSCs utilized for culture for bulk RNA seq and for in vivo experiments were used between passage 1–3. MSCs utilized for co‐culture with CRISPR‐edited cells were used between passage 2–5.

### Mouse models

NOD/SCID/*IL2rγ* ^−/−^/*IL3/GM/SF* (NSG‐SGM3, RRID: IMSR_JAX: 013062) mice were originally obtained from Leonard Shultz (The Jackson Laboratory) and bred at the Francis Crick Institute in the Biological Research facility. Both male and female mice between 8 and 14 weeks of age were used. Mice were bred. All in vivo studies were performed under project license number PP9619702, approved by UK Home Office and in accordance with The Francis Crick Institute Ethics guidelines.

### 3D scaffold in vivo experiment

The methodology used has been described in^20^ and^21^.

In brief, Gelfoam collagen‐based sponges (Cat 00300090315085; Pfizer) were cut into 24 smaller pieces of similar size (6.6 mm × 7.5 mm × 7 mm) by using a sterile scalpel. Spongostan sponges (Cat MS002; Ethicon) were also used for scaffolds pre‐seeded with CRISPR‐edited CD34^+^ HSPCs. Excised scaffolds were sterilized in 70% ethanol and then washed twice in phosphate‐buffered saline to ensure complete removal of the ethanol.

Subsequently, MSCs previously expanded where injected in scaffolds at a concentration of 0.5 × 10^5^ to 1 × 10^5^ cells in 50 µL/scaffold. MSC‐injected scaffolds were incubated in a plate for 1.5–2 h at 37 C. After 2 h, MSC media was gently added in each well containing hanging scaffolds and incubated overnight. The following day, 0.7 × 10^5^–1.2 × 10^5^ CD34^+^ HSPCs were injected in 50 µL/scaffold in Stem span SFEM II (Cat 09605; Stem Cell Technologies) with 1% of penicillin/streptomycin (Cat P4333; Sigma‐Aldrich), containing a cocktail of cytokines (20 ng/mL G‐CSF, 20 ng/mL IL‐3, 20 ng/mL TPO, Cat 300‐23, Cat 200‐03, Cat 300‐18, respectively; Peprotech). Scaffolds were then incubated between 1 and maximum 2 days before subcutaneous implantation of the scaffold in immunodeficient mice.

At the end of the experiment (10–12 weeks post‐implantation), mice were sacrificed, scaffolds retrieved and enzymatically digested (Dispase, DNAse I (Cat D4527‐500KU; Sigma) at 10 µg/mL, Collagenase type I (Cat C0130‐1G; Merck) at 1 mg/mL and 10% FBS (Sigma‐Aldrich). Digested scaffolds were then filtered through a sterile 5 mL tube with a cell strainer cap. After the first wash, supernatant was collected for secretome studies.

### Flow cytometry analysis and cell sorting for xenografted samples

Cells retrieved from the scaffolds at the end of the in vivo experiments were resuspended in 50 µL containing Fc Blocker (Cat 422302; BioLegend) and incubated for 10 min at RT. Cells were then stained with antibodies for human and murine antigens and incubated for 30 min at RT.

The antibodies used to analyze via FACS and cell sort xenografted cells are: hCD19‐FITC (clone HIB19, eBioscience, RRID: AB_1272 053; eBioscience), hCD33‐PE (clone WM53, RRID:AB_395843; BD Biosciences), hCD41‐APC (clone HIP8, AB_398671; BD Biosciences,), hCD45‐APCefluor780 (clone HI30, RRID: AB_1944368; eBioscience), hCD73‐BV605 (clone AD2, RRID:AB_2738063; BD Biosciences), mCD31‐PEcy7 (clone 390, AB_2716949; eBioscience), mCD45‐PERCPcy5.5 (clone 30‐F11, RRID:AB_1107 002; eBioscience). DAPI (4′,6‐diamidino‐2‐phenylindole, Cat D9542; Sigma‐Aldrich) staining was used to exclude dead cells.

Cells were analyzed as human hematopoietic cells (mCD45^−^hCD45^+^), myeloid cells (hCD45^+^hCD33^+^), B cells (hCD45^+^hCD33^−^hCD19^+^), MSCs (mCD45^−^hCD45^−^mCD31^−^hCD73^+^), and immature megakaryocytes (hCD45^+^hCD33^−^hCD19^−^hCD41^+^). Cells were sorted as mCD45^−^hCD45^+^.

### Luminex assay

Scaffold supernatants were probed for the following 44 analytes: CD40 Ligand, EGF, Eotaxin, FGF‐2, Flt‐3 Ligand, G‐CSF, GM‐SCF, Granzyme B, Groa, Grob, IFN‐α2, IFN‐β, IFN‐γ, IL‐1α, IL‐1β, IL‐1ra, IL‐2, IL‐3, IL‐4, IL‐5, IL‐6, IL‐7, IL‐8, IL‐10, IL‐12 p70, IL‐13, IL‐15, IL‐17A, IL‐17E, IL‐33, MCP‐1, MIP‐1α, MIP‐1β, MIP‐3α, MIP‐3β, PDGF‐AA, PDGF‐AB/BB, PD‐L1/B7‐H1, RANTES, TGF‐α, TNF‐α, TNF‐β, TRAIL, and VEGF.

The Human XL Cytokine Fixed Panel (Cat LKTM014; Biotechne) was performed following manufacturer's instructions. Supernatant samples were thawed and diluted 1:2. Samples were analyzed with the Bio‐Rad BioPlex 200 ELISA analyzer Luminex 200 (serial number LX10008283404).

### Bulk RNA sequencing

Sorted MSCs were pelleted, and RNA was extracted using an RNA purification kit (Zymo Research). RNA was quantified using Qubit (Invitrogen™ Qubit™ 3 Fluorometer). Total RNA quality was checked using Agilent 4200 TapeStation (Agilent Technologies). Samples with RNA integrity number (RIN) ≥8 were used for library preparation. Ribosomal RNA (rRNA) was depleted using the oligo d(T) approach: oligo(dT) primers used during the reverse transcription steps were able to capture only RNA with poly(A) tails. ~10 ng of RNA/sample were used for library preparation using NEBNext Ultra II Directional PolyA mRNA kit (New England Biolabs), following manufacturer's instructions. PCR products were purified following instructions above and library quality checked with Agilent Bioanalyser. After sequencing, FASTQ files were trimmed using TrimGalore v. 0.6.0 to remove adapters. Trimmed FASTQ files were aligned to the Homo sapiens genome using the GRCh38 reference genome. A pre‐built Salmon reference which uses selective alignment was downloaded from Refgenie and Salmon 1.9.0^22^ was used to quantify the sequencing reads using default paired‐end parameters. Transcript‐level quantification data was then loaded in R v. 4.3.2 using the tximport v. 1.30.0 R/Bioconductor package^23^ and summarized at gene level. A DESeq DataSet object was created including counts and metadata using the DESeq. 2 R/Bioconductor package v. 1.42.0,^24^ which was also used for Principal Component Analysis (PCA). Differential expression analyses were carried out using the limma‐voom framework in R^25^ using a design that takes in account the MPN type variable only and estimating the duplicate correlation between replicates MSCs from the same donor through the duplicateCorrelation function from limma. More specifically, the voom and duplicateCorrelation functions were used twice, with the second time using the consensus correlation estimated in the first time. log2(Fold Change) values were shrunk using the empirical Bayes method from limma. For pathway analysis, Gene Set Enrichment Analysis (GSEA) was performed using the fgsea R/Bioconductor package v. 1.28.0^26^ using as input genes ranked according to moderated log2(Fold Change) and run against gene sets from the Molecular Signature DataBase (MSigDB)^27^ using the C5 collection (Gene Ontology). All plots were created using the ggplot2 R package v. 3.4.4^28^ and the ComplexHeatmap package 2.18.0.^29^

### Statistical analysis

Statistical analyses were performed using GraphPad Prism Version 10 software (GraphPad). The specific statistical test used is indicated in each figure legend. In summary, for human engraftment analysis, one‐way ANOVA test, Mann–Whitney test, or Wilcoxon test were performed (specified in each figure legend). For percentage of human MSCs or mouse endothelial cells in scaffolds, Kruskal‐Wallis test was performed. For lineage distribution analysis, two‐way ANOVA test was performed. For the comparison of percentage of *JAK2* ^*V617F*^ mutation burden, Mann–Whitney test, paired *t* test, one‐way ANOVA test, or Kruskal–Wallis test were used.

Engraftment and cytokine concentration are presented as median ± interquartile range. Lineage distribution and percentage of *JAK2* ^*V617F*^ mutation burden are presented as mean ± SD. All significant *p*‐values are provided in the figure legends where applicable.

## RESULTS

### PV and ET BM patient‐derived MSCs can support healthy hematopoiesis in vivo

Investigating the transcriptomic signature of patient‐derived MSCs is ought to help uncover their activity in the development of PV and ET. Hence, we performed bulk RNA sequencing of MSCs derived from PV and ET BM and compared to MSCs derived from healthy donors (HD). In the Principal Component Analysis (PCA), the highest amount of variance (along PC1) was given by the difference between PV MSCs and the rest, while ET MSCs clustered more closely to HD MSCs (Supporting Information S1: Figure 1A). Gene Set Enrichment Analysis on differentially expressed genes between MSCs revealed no major differences between ET MSCs and HD MSCs, while as PV MSCs appeared to have upregulated pathways involved in DNA replication, proliferation and in immune modulation (Supporting Information S1: Figure 1B). We then decided to assess the functionality of MSCs derived from PV, ET patients, and HDs.

To evaluate the hematopoietic support provided by MSCs from PV and ET patients, we utilized a 3D scaffold humanized model in vivo, designed to recapitulate a more natural and supportive microenvironment for the engraftment and differentiation of human hematopoietic cells.^20^

Hence, we pre‐seeded scaffolds with MSCs from HD, PV or ET patients along with cord‐blood (CB) derived CD34^+^ HSPCs (Figure 1A, Supporting Information S1: Table 1 and Supporting Information S1: Supplementary Table 2). The results showed that healthy CB CD34^+^ HSPCs were able to engraft successfully in scaffolds containing HD, PV, or ET MSCs. Notably, CB CD34^+^ HSPCs engrafted similarly across all three types of scaffolds (Figure 1B and Supporting Information S1: Figure 1C). No anatomical differences were seen in scaffolds pre‐seeded with HD, PV, or ET MSCs (Figure 1C). Human CD45^+^ engraftment was independent of the age of the donor/patient MSCs pre‐seeded in the scaffolds (Supporting Information S1: Figure 1D). Additionally, no statistically significant differences were detected in the lineage distribution of the engrafted cells, with over 80% of the differentiated cells being CD33^+^ myeloid cells (Supporting Information S1: Figure 1E). We also performed FACS analysis on the stromal cells retrieved from these scaffolds. The percentage of MSCs (mCD45^‐^hCD45^‐^hCD73^+^hCD90^+^) retrieved from scaffolds seeded with PV MSCs were significantly decreased compared to HD or ET MSCs pre‐seeded scaffolds (Figure 1D). No difference on the percentage of murine endothelial cells (mCD45^−^hCD45^−^CD31^+^) could be seen (Figure 1E). Nevertheless, to fully define the differentiation potential of the human MSC‐derived progenies present in the scaffold post‐transplant from the different groups, will warrant further analysis.

Overall, similar levels of engraftment of CB CD34^+^ HSPCs in HD, PV, and ET MSCs pre‐seeded scaffolds suggest that MPN MSCs are still able to support healthy hematopoiesis, under these in vivo conditions.

### PV and ET BM patient‐derived MSCs can support CRISPR‐edited *JAK2* ^*V617F*^ HSPCs

After confirming that essential functions of PV and ET MSCs are preserved, we sought to explore their ability to sustain *JAK2* ^*V617F*^ HSPCs. To achieve this, we utilized the CRISPR‐Cas9 system to genetically edit the JAK2 locus in CB CD34^+^ HSPCs, as published in Baik et al. study.^30^ CRISPR‐edited *JAK2* ^*V617F*^ CD34^+^ HSPCs were then used in both ex vivo and in vivo humanized settings (Figure 1F).

We co‐cultured CRISPR‐edited *JAK2* ^*V617F*^ CD34^+^ HSPCs in vitro with either HD, PV, or ET (MPN) MSCs. Since one of the main features of MPN pathogenesis is the presence of an inflammatory milieu,^31^ we also investigated whether the presence of inflammatory stimuli, like lipopolysaccharide (LPS) and interleukin‐1 β (IL‐1β) stimulation, could promote the proliferation of CRISPR‐edited *JAK2* ^*V617F*^ clones.

To assess the *JAK2* ^*V617F*^ mutation burden, we performed next‐generation MiSeq sequencing at baseline (Day 0) and at the end of the co‐culture period. Interestingly, HD and MPN MSCs demonstrated the ability to maintain CRISPR‐edited *JAK2* ^*V617F*^ cells both at steady state and in presence of inflammatory stimuli, observed at both 1 week (Supporting Information S1: Figure 1F) and 2 weeks of co‐culture (Supporting Information S1: Figure 1G).

Next, we evaluated the hematopoietic support provided by HD and MPN MSCs to the CRISPR‐edited CD34^+^ HSPCs in our humanized 3D scaffold model in vivo (Figure 1F). Consistent with our in vitro findings, both HD and MPN MSCs successfully supported the engraftment of CRISPR‐edited cells (Figure 1G). Notably, the *JAK2* ^*V617F*^ mutation burden was maintained for 12 weeks following scaffold implantation, and even significantly increased in the presence of MPN stromal cells (Figure 1H). No differences in lineage distribution were observed between scaffolds pre‐seeded with HD or MPN MSCs (Supporting Information S1: Figure 1H).

### Primary PV and ET CD34^+^ HSPCs robustly engraft in a humanized 3D scaffold model in vivo

Our findings using the in vivo CRISPR model led us to investigate whether CD34^+^ HSPCs derived from PV and ET patients with *JAK2* ^*V617F*^ mutation could engraft in 3D scaffolds containing MSCs from PV and ET patients, respectively. We tested eight samples from PV patients and eight samples from ET patients (Figure 2A and Supporting Information S1: Table 3). Both PV and ET CD34^+^ HSPCs demonstrated successful engraftment in our 3D scaffold model (Figure 2B). While ET CD34^+^ HSPCs appeared to engraft slightly less effectively than their PV counterparts, this difference was not statistically significant (Figure 2B). Human CD45^+^ engraftment was independent of the age of the patient MSCs pre‐seeded in the scaffolds (Supporting Information S1: Figure 2A). Both PV and ET scaffolds exhibited a CD33^+^ myeloid expansion (Supporting Information S1: Figure 2B). To assess if our humanized model maintains the disease clonality, we FACS‐sorted the human CD45^+^ (hCD45^+^) cells and performed next‐generation MiSeq sequencing to evaluate and compare the *JAK2* ^*V617F*^ mutation burden in the primary sample (CD34^+^ HSPCs Day 0) and at the end of the in vivo experiment (Figure 2A). Both PV and ET humanized scaffolds maintained the *JAK2* ^*V617F*^ variant allele frequency (VAF), suggesting that a humanized niche is essential for the consistent engraftment of primary CD34^+^ HSPCs from PV and ET patients (Figures 2C,D, respectively). Additionally, we also investigated if MPN CD34^+^ HSPCs engrafted in the humanized niche could colonize the murine BM and spleen. We report that no hCD45^+^ cells were detected, suggesting that human cells engraft and stay in the scaffold structures (Figure 2E,F). The behavior of MPN HSPCs is similar to evidence that has been previously observed in MDS HSPC xenotransplantation model.^21^

### Primary PV and ET CD34^+^ HSPCs can be sustained by both HD and PV or ET BM MSCs in vivo

Understanding the stromal dependency of CD34^+^ HSPCs in PV and ET is crucial for uncovering insights into the interactions between PV and ET mutant clones with the BM niche. To explore this, we conducted an in vivo experiment in which we implanted scaffolds pre‐seeded with a pooled population of PV or ET patient CD34^+^ HSPCs, alongside either HD MSCs or PV or ET MSCs, respectively (Figure 3A). Interestingly, scaffolds pre‐seeded with PV CD34^+^ HSPCs exhibited similar levels of hCD45^+^ engraftment, regardless of whether they were pre‐seeded with HD or PV MSCs (Figure 3B). We also assessed the *JAK2* ^*V617F*^ VAF in hCD45^+^ cells that were FACS‐sorted from scaffolds containing either HD or PV MSCs at the end of the in vivo experiment. The mutation burden was compared to that at Day 0 of the in vivo experiment, which corresponds to the mutation burden found in the primary patient samples. In PV scaffolds, the *JAK2* ^*V617F*^ VAF was maintained in both HD and PV MSC‐pre‐seeded scaffolds when compared to day 0 levels (Figure 3C).

Similarly, scaffolds containing ET CD34^+^ HSPCs showed comparable human CD45^+^ engraftment levels between those pre‐seeded with HD or ET MSCs (Figure 3D). The *JAK2* ^*V617F*^ VAF was generally maintained across both HD and ET MSC‐pre‐seeded scaffolds, with a slight trend towards a higher *JAK2* ^*V617F*^ mutation burden in the ET MSC‐pre‐seeded scaffolds, though this result did not reach statistical significance (Figure 3E). There were no noticeable differences in lineage distribution between the ET and PV scaffolds when comparing scaffolds with either HD, PV or ET stroma, with over 80% of the human cell population being hCD33^+^ myeloid (Supporting Information S1: Figure 2C). No anatomical differences were seen in scaffolds pre‐seeded with HD or PV or ET MSCs (Supporting Information S1: Figure 2D). Human engraftment was independent of the age of the donor/patient MSCs pre‐seeded in the scaffolds (Supporting Information S1: Figure 2E and Supporting Information S1: Figure 2F).

### Secretome profiling in PV and ET scaffolds can reveal interactions between mutant clones and the BM microenvironment

To gain insights into the interactions of MSCs and HSPCs, we performed a secretome analysis from the scaffolds seeded with either HD or MPN stroma. We used a Luminex assay on the supernatants collected from 12 weeks postimplanted scaffolds (Figure 3A). We tested a panel of 44 analytes, identifying several cytokines that were consistently detected in all scaffolds analyzed, including fibroblast growth factor 2 (FGF‐2), granulocyte‐macrophage colony‐stimulating factor (GM‐CSF), IL‐1β, interleukin‐1 receptor antagonist (IL‐1ra), interleukin 8 (IL‐8), and monocyte chemoattractant protein‐1 (MCP‐1) (Supporting Information S1: Figures 2G–L). We found no major differences in cytokine concentrations between PV and ET scaffolds pre‐seeded with HD, PV, or ET stroma, respectively. GM‐CSF concentration was found at higher levels in scaffolds pre‐seeded with MPN (PV or ET) MSCs (Supporting Information S1: Figure 2H). However, it was not possible to determine if this cytokine was secreted by human cells seeded in the scaffolds, or by the humanized mice, making the interpretation and conclusions difficult. Nevertheless, when analysing ET CD34^+^ HSPCs pre‐seeded scaffolds, FGF‐2 was detected in both HD MSC and ET MSC pre‐seeded scaffolds, with a slight trend towards higher concentrations in scaffolds pre‐seeded with ET MSCs (Supporting Information S1: Figure 3A). FGF‐2 is known to be secreted by MSCs and plays a critical role in promoting the proliferation of megakaryocyte progenitors and the maturation of megakaryocytes in the BM.^32^ Importantly, the concentration of FGF‐2 in the ET scaffolds correlated with the number of MSCs retrieved (Pearson *r* = 0.64, *p* = 0.004) (Figure 3F), following xenotransplantation. However, no correlation between FGF‐2 and MSC cell numbers was noticed in PV scaffolds (Supporting Information S1: Figure 3B). Furthermore, in ET scaffolds, FGF‐2 did not correlate to human engraftment, suggesting that human MSCs might be the main source of FGF‐2 (Supporting Information S1: Figure 3C). None of the other cytokines detected correlated with MSC cell numbers retrieved in both PV or ET conditions (Supporting Information S1: Figures 3D–H). Intriguingly, higher levels of FGF‐2 were associated with scaffolds exhibiting a greater *JAK2* ^*V617F*^ VAF, suggesting that FGF‐2 secreted by the MSCs in contact with ET CD34^+^ HSPCs may contribute to the *JAK2* ^*V617F*^ clonal expansion (Pearson *r* = 0.91, *p* = 0.0002) (Figure 3G). Moreover, the number of MSCs recovered from ET scaffolds correlated with elevated levels of *JAK2* ^*V617F*^ mutation burden (Pearson *r* = 0.78, *p* = 0.007) (Figure 3H). Given that ET is characterized by an overproduction of megakaryocytes, we further investigated the presence of a human CD41^+^ population in scaffolds pre‐seeded with primary ET CD34^+^ HSPCs (Supporting Information S1: Figure 3I). Scaffolds pre‐seeded with ET CD34^+^ HSPCs indeed contained a hCD45^+^hCD41^+^hCD33^−^hCD19^−^ population. Notably, scaffolds pre‐seeded with ET MSCs were associated with a higher percentage of the hCD45^+^hCD41^+^hCD33^−^hCD19^−^ population (Figure 3I) in the majority of cases, compared to scaffolds pre‐seeded with HD MSCs.

## DISCUSSION

A substantial amount of evidence underscores the critical role of the BM niche—particularly the BM stroma—in supporting the survival of tumor cells, shielding mutant clones from chemotherapy, and promoting a fibrotic environment alongside inflammation.^33^ The interplay between the microenvironment and malignant cells is pivotal in understanding the progression of diseases such as MPN. However, one of the significant challenges in studying MPN lies in the absence of a reliable in vivo model to examine human primary MPN HSPCs.

Traditional xenograft models often yield low engraftment levels as well as require a substantial number of injected cells^16^ and lack a human microenvironment, limiting interaction analysis between human primary MPN cells and stroma.

In this study, we employed a humanized 3D scaffold model^20^ to investigate the supportive role of stromal cells derived from MPN, specifically PV and ET MSCs, on primary MPN CD34^+^ HSPCs. First, we investigated potential transcriptomic signatures of PV and ET MSCs compared to HD MSCs. Consistent with Chen et al., we noticed an increased proliferation pathways in PV MSCs compared to HD.^34^ However, this approach consisted in sequencing expanded MSCs, which may not fully represent the native signature present in PV and ET BM. Then, we provided in vivo evidence that PV and ET BM MSCs support healthy hematopoiesis at levels comparable to those of HD MSCs. Although HD MSCs used were from young adults (21–31 years of age), ET and PV MSCs were able to robustly support HD CD34^+^ HSPCs in vivo, despite the potential biological disadvantage posed by ageing. This indicates no significant dysfunctional phenotypes in the PV and ET MSCs. Therefore, these results suggest that MSCs retain their supportive role in hematopoiesis, even within the pathological context of MPN.

Before assessing the hematopoietic support of PV and ET MSCs towards their respective CD34^+^ HSPCs in primary patient samples, we initially evaluated the hematopoietic support provided by MPN stroma to a *JAK2* ^*V617F*^ CRISPR model, as *JAK2* ^*V617F*^ mutation is the most common mutation found in MPN patients.^1^ Due to our limited availability of HSPCs from HD BM samples and taking in consideration that *JAK2* ^*V617F*^ mutation can be detected as early as the in‐utero stage,^35^ we used CD34^+^ HSPCs extracted from CB. In vitro data demonstrated that HD and PV/ET MSCs can maintain *JAK2* ^*V617F*^ cells over time; however, LPS and IL‐1β did not provide proliferative advantage to the mutant clones. The proliferative advantage of the *JAK2* clone may be specifically supported by other inflammatory stimuli. In fact, different types of inflammation have been shown to support specific mutant clones, depending on the nature of the HSC mutation.^36^ Our in vivo data confirmed the capacity of HD and PV/ET MSCs to sustain CRISPR‐edited *JAK2* mutated cells. However, it would be valuable to validate these findings in HD BM samples. The increased *JAK2* ^*V617F*^ mutation burden observed in MPN MSC‐seeded scaffolds suggest that the MPN niche may foster conditions that are favorable for mutant clone expansion.

Our initial findings prompted us to test primary BM PV and ET CD34^+^ HSPCs in 3D scaffolds in vivo. Remarkably, both HD and PV or ET MSCs were able to sustain reliable human cell engraftment and maintain the *JAK2* ^*V617F*^ mutation burden in vivo, suggesting that PV and ET CD34^+^ HSPCs rely on human stroma for their survival and proliferation. No significant lineage skewing was observed, as a predominant myeloid bias was present across all groups. However, the pronounced myeloid expansion in our model may limit the ability to detect subtle differences in lineage distribution.

The similarity in engraftment levels between HD, PV, and ET MSC‐seeded scaffolds reinforces the notion that PV and ET MSCs retain essential functionality within the BM niche. This finding is consistent with studies showing that the BM microenvironment in myeloid malignancies plays a key role in supporting the survival and proliferation of mutant clones.^21^ Future work needs to focus on dissecting the stromal heterogeneity and explore the crosstalk with the mutant clones by developing techniques to enable 3D imaging of scaffolds and single‐cell RNA sequencing (scRNAseq) on stroma cells retrieved from the scaffolds.

To gain insights into the interactions between MSCs and HSPCs, we performed a secretome analysis from the scaffolds. This analysis revealed interesting findings regarding cytokine signaling within the MPN BM niche. In ET scaffolds, FGF‐2 levels correlated with the number of MSCs retrieved, increased frequency of CD41^+^ expressing cells as well as higher *JAK2* ^*V617F*^ mutation burden, suggesting a possible role of FGF‐2 in promoting the expansion of *JAK2* ^*V617F*^ mutant clones. Elevated levels of FGF‐2 have been previously reported in different hematological cancers, such as acute myeloid leukemia (AML),^37^ myelodysplastic syndromes (MDS) as well as MPN^38^ and is associated with poorer prognosis in non‐Hodgkin's lymphoma.^39^ Further in‐depth analysis is needed to reveal whether FGF‐2 is indeed directly involved in the growth and differentiation of ET HSPCs, which may suggest a new avenue for targeted interventions in MPN.

In conclusion, this new MPN in vivo model will enable a more robust preclinical exploration of the interactions between human stroma and primary MPN cells, which will lead to the identification of new targets involved in sustaining the growth of mutant clones.

## AUTHOR CONTRIBUTIONS

**Alessandra Ferrelli**: Investigation; methodology; writing—original draft; writing—review and editing; formal analysis; data curation. **Syed Mian**: Investigation; methodology; writing—review and editing; formal analysis. **Marion Piganeau**: Investigation; methodology. **Hector Huerga Encabo**: Investigation; writing—review and editing; methodology; formal analysis. **Despoina Papazoglou**: Investigation; methodology; validation; formal analysis. **Giuseppe D'Agostino**: Investigation; methodology; visualization; writing—review and editing; formal analysis; data curation. **Fatihah Mohamad Nor**: Investigation; methodology; validation; formal analysis; writing—review and editing. **Manuel Garcia‐Albornoz**: Investigation; methodology; visualization; formal analysis; data curation; writing—review and editing. **Steven Ngo**: Investigation; methodology; validation; formal analysis; writing—review and editing. **Linda Ariza‐McNaughton**: Investigation; validation; methodology; formal analysis; writing—review and editing. **Erika Morsia**: Writing—review and editing; resources. **Matteo Giovanni Della Porta**: Investigation; resources; writing—review and editing. **Claire Harrison**: Conceptualization; writing—review and editing. **Shahram Kordasti**: Conceptualization; funding acquisition; project administration; supervision; writing—review and editing; resources. **Dominique Bonnet**: Supervision; formal analysis; writing—review and editing; writing—original draft; conceptualization; investigation; resources.

## CONFLICT OF INTEREST STATEMENT

The authors declare no conflict of interest.

## FUNDING

This work was supported by the Francis Crick Institute, which receives its core funding from Cancer Research UK (CC2027), the UK Medical Research Council (CC2027), and the Wellcome Trust (CC2027) to DB. AF was supported by the Cancer Research UK, City of London Centre PhD fellowship (CTRQQR‐2021\100004). HHE was supported by the Kay Kendall fellowship (KKL1397). MGDP was supported by AIRC Foundation (Associazione Italiana per la Ricerca contro il Cancro, Milan, Italy—projects 29483 and 21267). SK was supported by Blood Cancer UK grant ref. 22009 and US DoD Award W81XWH‐22‐1‐1097, BM210014.

## Supporting information

Supplementary 1.

Supplementary 2.

Supplementary 3.

Supplementary Table 1.

Supplementary Table 2.

Supplementary Table 3.

Supplementary Table 4.

Supplementary Table 5.

Supplementary figure and table legends ‐ new changes in green.

## Data Availability

The data that supports the findings of this study is available in the supplementary material of this article. Bulk RNA sequencing raw and processed files have been deposited at GEO (accession number: GSE288706), and will be publicly available at the date of the publication of this paper. Any additional information required to reanalyze the data reported in this paper will be available from the lead contact upon request.
