# Supplementary figures and images for: Mesenchymal stromal cells from JAK2 V617F myeloproliferative neoplasms support healthy and malignant hematopoiesis in a humanized scaffold model in vivo
Source: Hemasphere. 2025 Aug 22;9(8):e70185. doi: 10.1002/hem3.70185 (PMC12374162; doi:10.1002/hem3.70185)

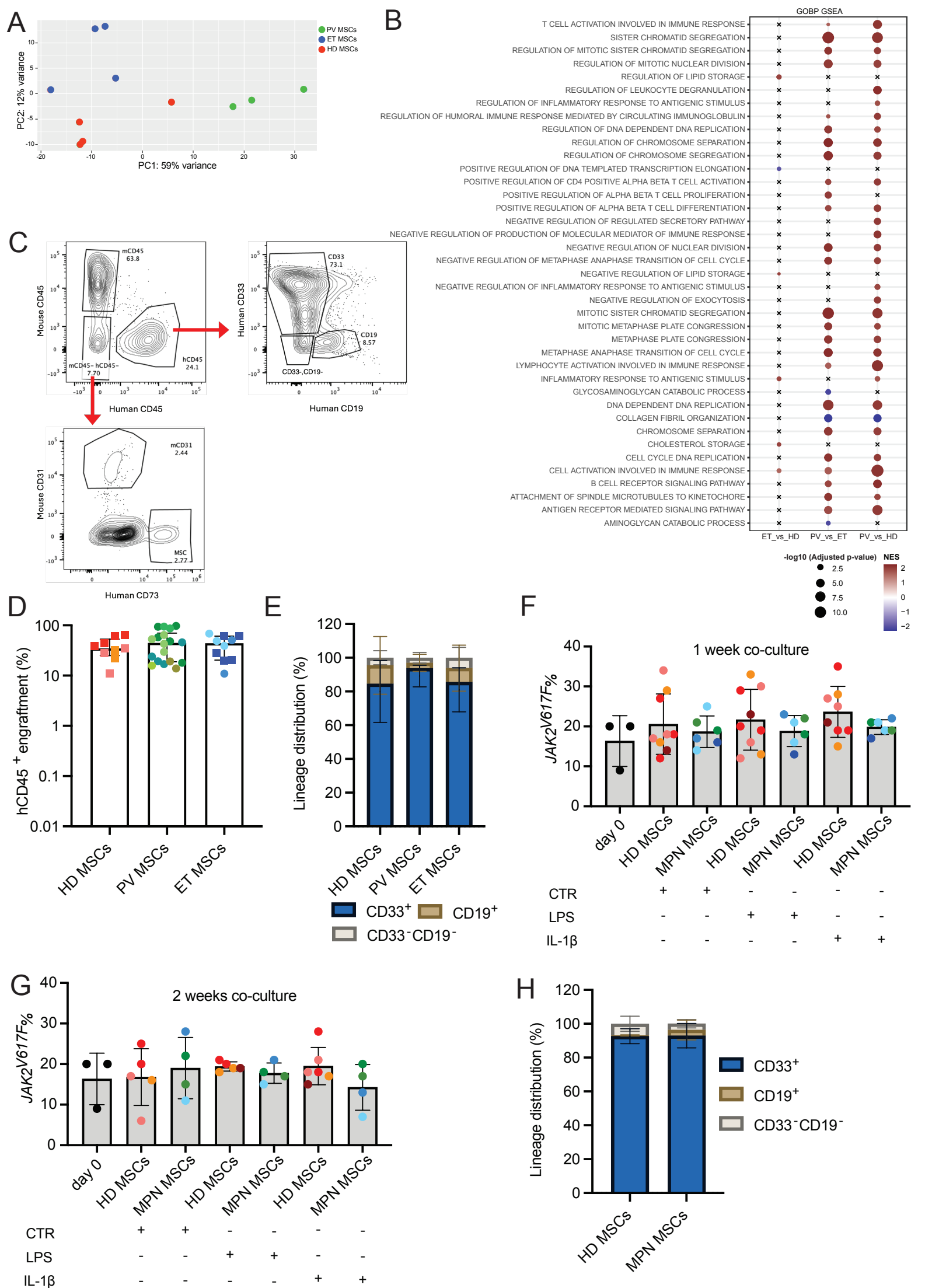

Supplement: Supplementary file 1 — Supplementary 1. [file HEM3-9-e70185-s004.pdf]

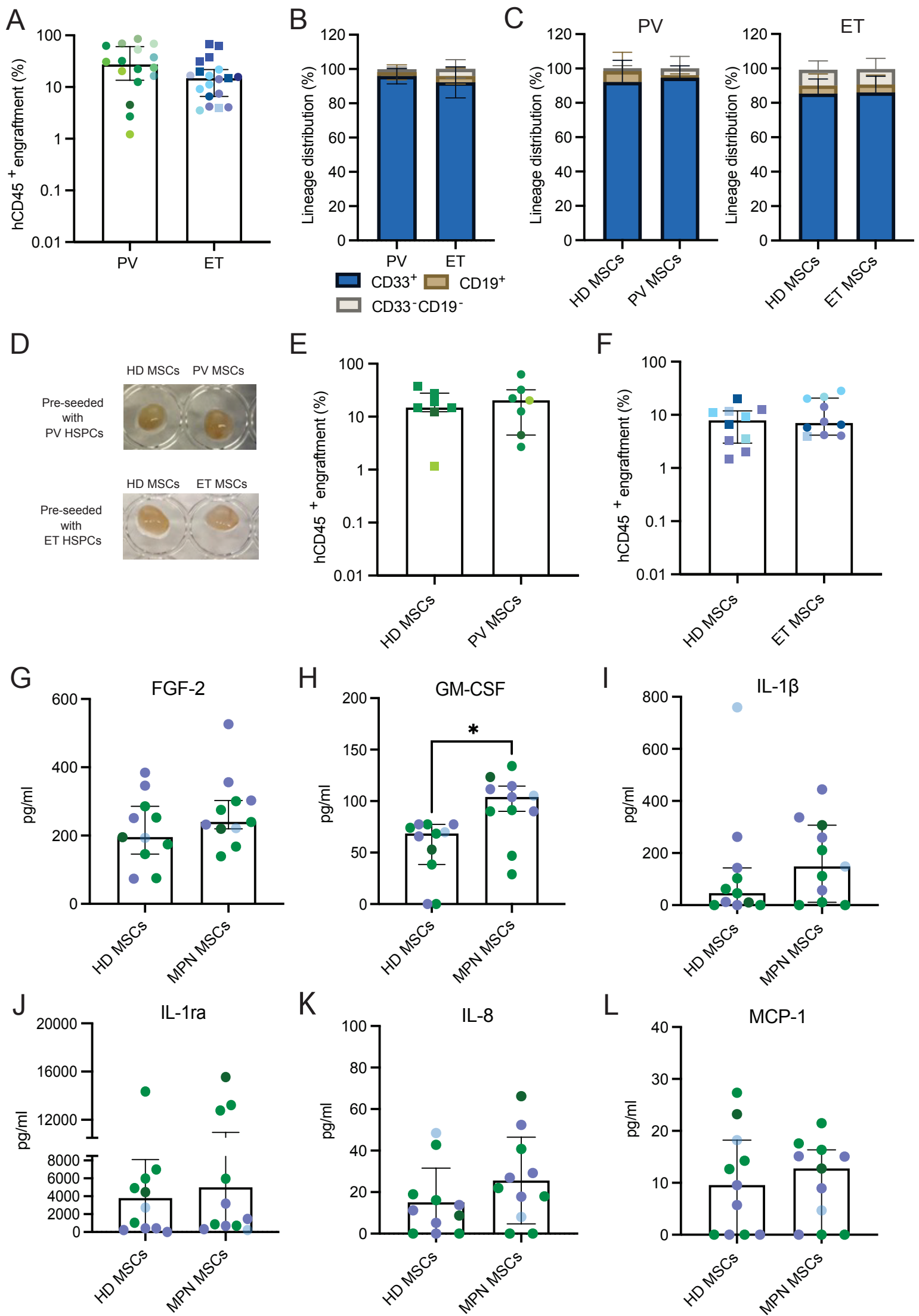

Supplement: Supplementary file 2 — Supplementary 2. [file HEM3-9-e70185-s003.pdf]

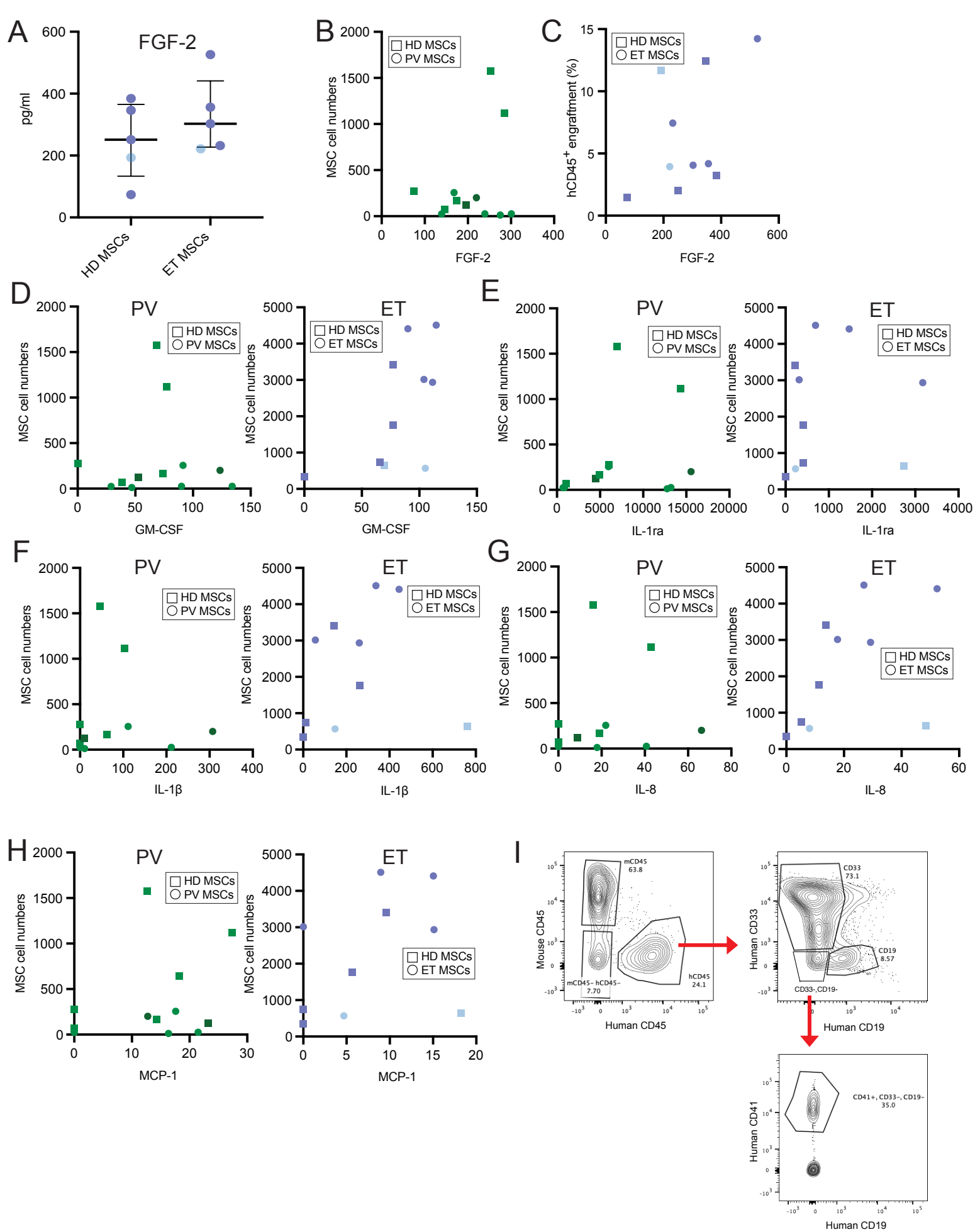

Supplement: Supplementary file 3 — Supplementary 3. [file HEM3-9-e70185-s008.pdf]
