# Supplementary material for: Mesenchymal stromal cells from JAK2 V617F myeloproliferative neoplasms support healthy and malignant hematopoiesis in a humanized scaffold model in vivo
Source: Hemasphere. 2025 Aug 22;9(8):e70185. doi: 10.1002/hem3.70185 (PMC12374162; doi:10.1002/hem3.70185)
Supplement: Supplementary file 9 — Supplementary figure and table legends ‐ new changes in green. [file HEM3-9-e70185-s001.docx]

**Supplementary Figure legends**

**Supplementary Figure 1**

1. Principal Component Analysis (PCA) of PV, ET and HD MSCs cultured alone showing the first two principal components. MSCs were used between passage 1-3.
2. Gene Set Enrichment Analysis for differential expression analysis comparing ET MSCs to HD MSCs, PV MSCs to HD MSCs, and PV MSCs to ET MSCs. Gene sets from Gene Ontology – Biological Process significant in at least one comparison. NES: normalised enriched score.
3. Representative FACS plot of human CD45^+^ cell engraftment and gating strategy of humanised scaffolds after 11-12 weeks after scaffold implantation in NSG-SGM3 mice.
4. Engraftment of HD HSPCs in scaffolds pre-seeded with HD, PV or ET MSCs (data from Figure 1A,1B,1C). Donor/Patient MSCs that are <40 years old have been labelled as ‘square’, while all the other points (round dots) have MSCs with >40 years old as mean age. Error bars: median ± interquartile range.
5. Lineage distribution within the hCD45^+^ cells in humanised scaffolds pre-seeded with either HD, ET or PV MSCs and hCD34^+^ HSPCs. Data representative of 3 independent experiments, each performed with hCD34^+^ HSPCs from different human umbilical cord blood donors. Error bars: mean ± SD. 2-way Anova test used for significance (non-significant).
6. Percentage of *JAK2^V617F^* mutation burden in CRISPR-edited CD34^+^ HSPCs at day 0 and in hCD45^+^ cells after 1-week co-culture at steady stare or stimulated with either 1 μg/ml LPS or with 1ng/ml IL-1β with either HD or MPN MSCs used as stromal layer. Error bars: mean ± SD. Data representative of 3 independent experiments, each performed with hCD34^+^ HSPCs from different human umbilical cord blood donors. Each pool of patient CD34^+^ HSPCs are colour coded. Ordinary one-way Anova test used for significance (non-significant).
7. Percentage of *JAK2^V617F^* mutation burden in CRISPR-edited CD34^+^ HSPCs at day 0 and in hCD45^+^ cells after 2-week co-culture at steady stare or stimulated with either 1 μg/ml LPS or with 1ng/ml IL-1β with either HD or MPN MSCs used as stromal layer. Error bars: mean ± SD. Data representative of 3 independent experiments, each performed with hCD34^+^ HSPCs from different human umbilical cord blood donors. Each pool of patient CD34^+^ HSPCs are colour coded. Ordinary one-way Anova test used for significance (non-significant).
8. Lineage distribution within the hCD45^+^ cells in humanised scaffolds pre-seeded with either HD, ET or PV MSCs and CRISPR-edited CD34^+^ HSPCs.

**Supplementary Figure 2**

1. Engraftment of PV and ET HSPCs in scaffolds pre-seeded with PV or ET MSCs, respectively (data from Figure 2B). Patient MSCs that are <40 years old have been labelled as ‘square’, while all the other points (round dots) have MSCs with mean age >40 years old. Error bars: median ± interquartile range.
2. Lineage distribution within the hCD45^+^ cells in humanised scaffolds pre-seeded with either PV or ET MSCs and PV or ET hCD34^+^ HSPCs, respectively. Error bars: mean ± SD. 2-way Anova used for significance (non-significant).
3. Lineage distribution within the hCD45^+^ cells in humanised scaffolds pre-seeded with either HD or PV MSCs and PV CD34^+^ HSPCs (left graph) and with either HD MSCs or ET MSCs (right graph). Error bars: mean ± SD. Legend can be found in Supplementary Figure 2B.
4. Photo of representative scaffolds pre-seeded with HD or PV MSCs and PV CD34^+^ HSPCs and scaffolds pre-seeded with HD or ET MSCs and ET CD34^+^ HSPCs retrieved at 12 weeks from *in vivo* implantation.
5. Engraftment of PV HSPCs in scaffolds pre-seeded with HD or PV MSCs, respectively (data from Figure 3B). Donor/Patient MSCs that are <40 years old have been labelled as ‘square’, while all the other points (round dots) have MSCs with >40 years old as mean age.
6. Engraftment of ET HSPCs in scaffolds pre-seeded with HD or ET MSCs, respectively (data from Figure 3D). Donor/Patient MSCs that are <40 years old have been labelled as ‘square’, while all the other points (round dots) have MSCs with >40 years old as mean age. Error bars: median ± interquartile range.
7. FGF-2 release from scaffolds implanted with PV or ET CD34^+^ HSPCs pre-seeded with either HD or PV and ET MSCs, respectively. Each pool of patient CD34^+^ HSPCs are colour coded (blue: ET and green: PV). Error bars: median ± interquartile range.
8. GM-CSF release from scaffolds implanted with PV or ET CD34^+^ HSPCs pre-seeded with either HD or PV and ET MSCs, respectively. Each pool of patient CD34^+^ HSPCs are colour coded (blue: ET and green: PV). Error bars: median ± interquartile range.
9. IL-1β release from scaffolds implanted with PV or ET CD34^+^ HSPCs pre-seeded with either HD or PV and ET MSCs, respectively. Each pool of patient CD34^+^ HSPCs are colour coded (blue: ET and green: PV). Error bars: median ± interquartile range.
10. IL-1ra release from scaffolds implanted with PV or ET CD34^+^ HSPCs pre-seeded with either HD or PV and ET MSCs, respectively. Each pool of patient CD34^+^ HSPCs are colour coded (blue: ET and green: PV). Error bars: median ± interquartile range.
11. IL-8 release from scaffolds implanted with PV or ET CD34^+^ HSPCs pre-seeded with either HD or PV and ET MSCs, respectively. Each pool of patient CD34^+^ HSPCs are colour coded (blue: ET and green: PV). Error bars: median ± interquartile range.
12. MCP-1 release from scaffolds implanted with PV or ET CD34^+^ HSPCs pre-seeded with either HD or PV and ET MSCs, respectively. Each pool of patient CD34^+^ HSPCs are colour coded (blue: ET and green: PV). Error bars: median ± interquartile range.

**Supplementary Figure 3**

1. FGF-2 release from scaffolds implanted with ET CD34^+^ HSPCs pre-seeded with either HD or ET MSCs. Each pool of patient CD34^+^ HSPCs is colour coded. Error bars: median ± interquartile range.
2. Correlation between MSC cell numbers retrieved from PV scaffolds and FGF-2 release from the scaffolds. Each pool of patient CD34^+^ HSPCs is colour coded. Correlation was measured by linear regression analysis.
3. Correlation between human engraftment in ET scaffolds and FGF-2 release in the scaffolds. Each pool of patient CD34^+^ HSPCs is colour coded. Correlation was measured by linear regression analysis.
4. Correlation between MSC cell numbers retrieved from PV or ET scaffolds and GM-CSF release from the scaffolds. Each pool of patient CD34^+^ HSPCs is colour coded. Correlation was measured by linear regression analysis.
5. Correlation between MSC cell numbers retrieved from PV or ET scaffolds and IL-1ra release from the scaffolds. Each pool of patient CD34^+^ HSPCs is colour coded. Correlation was measured by linear regression analysis.
6. Correlation between MSC cell numbers retrieved from PV or ET scaffolds and IL-1β release from the scaffolds. Each pool of patient CD34^+^ HSPCs is colour coded. Correlation was measured by linear regression analysis.
7. Correlation between MSC cell numbers retrieved from PV or ET scaffolds and IL-8 release from the scaffolds. Each pool of patient CD34^+^ HSPCs is colour coded. Correlation was measured by linear regression analysis.
8. Correlation between MSC cell numbers retrieved from PV or ET scaffolds and MCP-1 release from the scaffolds. Each pool of patient CD34^+^ HSPCs is colour coded. Correlation was measured by linear regression analysis.
9. Representative FACS plot of gating strategy for hCD45^+^CD41^+^ cells and hCD45^+^CD33^-^CD19^-^CD41^+^ cells after 11-12 weeks after scaffold implantation in NSG-SGM3 mice.

**Supplementary Table legends**

**Table S1.** Summary list of all patient samples used in this study.

**Table S2.** List of patient samples used for experiments presented in Figure 1.

**Table S3.** List of patient samples used for experiments presented in Figure 2.

**Table S4.** List of patient samples used for experiments presented in Figure 3.

**Table S5.** Sequences of small guide RNA, donor DNA template and primers for *JAK2*.
